# Single-Port Robotic Liver Surgery: A Pilot Feasibility Study of a Standardized Surgical Approach

**DOI:** 10.3390/jcm15135028

**Published:** 2026-06-27

**Authors:** Silvio Caringi, Antonella Delvecchio, Annachiara Casella, Valentina Ferraro, Francesca Romano, Matteo Stasi, Nunzio Tralli, Susana Abigail Diaz Menjivar, Henriquez Angel, Riccardo Memeo, Michele Tedeschi

**Affiliations:** 1 Unit of Hepato-Biliary and Pancreatic Surgery, “F. Miulli” General Hospital, 70021 Acquaviva delle Fonti, Italy; 2 Department of Medicine and Surgery, LUM University, 70010 Casamassima, Italy; 3 Department of General Surgery, Hospital Nacional Rosales of El Salvador, National University of El Salvador, San Salvador 1101, El Salvador; 4 Department of Digestive Surgery, Avante Hospital Complex, San Salvador 1101, El Salvador; ahenriquez@complejoavante.com

**Keywords:** single-port robotic surgery, robotic liver resection, minimally invasive liver surgery, robotic hepatectomy, enhanced recovery

## Abstract

**Background**: Minimally invasive liver surgery has continuously developed with the advent of robotic systems that could present some advantages regarding dexterity and visualization. Single-port robotic devices have been introduced more recently in order to minimize the invasiveness of surgery. Unfortunately, scientific literature on this topic is still poor. This pilot feasibility study aimed to assess the technical applicability and short-term outcomes of single-port robotic liver resection. **Methods**: The study was designed as a retrospective analysis of 11 consecutive patients treated with single-port robotic liver resection. All interventions were performed in order to treat lesions localized in the anterolateral segments of the liver. All preoperative, intraoperative, and postoperative data were collected retrospectively and considered for the analysis. Cirrhotic patients were graded according to the Child–Pugh score. **Results**: The median age was 67 years (IQR 41–78), and 63.6% of the patients were women. There was cirrhosis in 27.3% of the cases, and all cases were categorized as Child–Pugh class A. Neoadjuvant chemotherapy was not administered in any of the patients. All procedures were considered Tampa grade II. The median operation time was 190 min (IQR 70–320), and the median blood loss was 50 mL (IQR 0–300). Pedicle clamping was done in 36.4% of the cases. An additional assistant trocar was needed in 45.4% of the procedures. In total, two anatomical and nine non-anatomical resections were done. There were no postoperative complications, reinterventions, and 90-day readmissions. The median length of hospitalization was 2 days (IQR 1–3). The postoperative pain was minimal, with a median VAS and NRS score of 0 on postoperative days 0 and 1. Analgesic treatment was ceased on postoperative day 1, and the median time to first flatus was 1 day in all patients. **Conclusions**: Single-port robotic liver resection seems to be technically possible in selected patients with intermediate-difficulty lesions in anterolateral segments. Additional research is necessary to establish its role in minimally invasive liver surgery.

## 1. Introduction

The constant advancements in minimally invasive surgery (MIS) have resulted in the creation of sophisticated robotic systems designed to minimize surgical trauma while maintaining precision in operation. The da Vinci Single-Port (SP) system can be described as the most recent innovation in robotic surgery, offering the possibility of using multiple fully articulated instruments and a three-dimensional flexible camera in one incision [1]. Such an approach was developed to address the shortcomings associated with multiport robotic surgery, such as instrument collision and limited maneuverability in small areas [2].

The SP system was initially used in urology and transoral surgery, and now, with more and more proof of the safety and technical success of SP operations in general surgery, the application of SP robots is becoming increasingly popular [3,4]. The latest trend associated with the use of SP robotics is the attempt to apply it to hepatobiliary surgery, which poses additional technical challenges due to the complexity of parenchymal transection and vascular control in the area [5,6].

## 2. Material and Methods

The current study is a retrospective case series of 11 consecutive patients undergoing robotic liver resection with the da Vinci SP robot at a single tertiary referral center from March 2025 to September 2025. It should be noted that all operations in the current study were performed by the same surgeon, thus providing standardization of the surgical technique in all cases, in a high-volume hepatobiliary center. The operating surgeon has mastered the learning curve for both laparoscopic and multiport robotic liver surgery prior to the adoption of the single-port platform and the institution performs more than 150 liver resections per year, of which 90% are minimally invasive.

**Figure 1:**
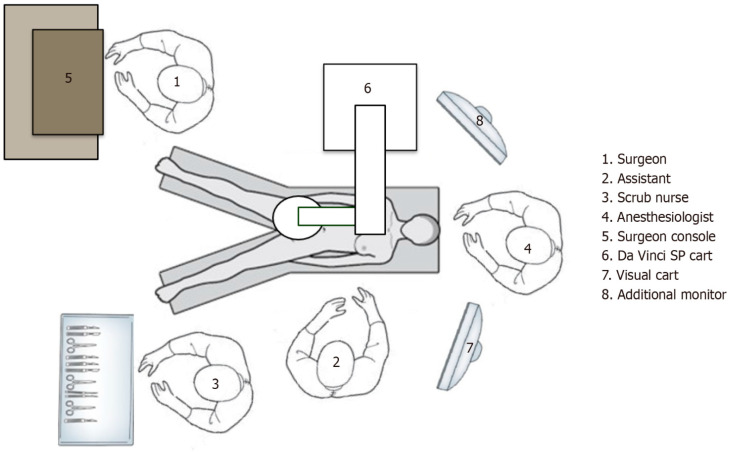
Operating Room setup. Patients were positioned in a supine position with a 15° reverse Trendelenburg inclination and 5° left rotation of the body.

Patients for inclusion in the current study had a solitary, resectable liver lesion localized in the anterolateral segments. Surgical indications encompassed both primary and secondary cancers of the liver such as hepatocellular carcinoma (36.4%) and liver metastasis from colorectal (45.4%) and breast cancer (18.2%) respectively. Inclusion criteria were: solitary lesion, location in anterolateral segments, absence of major vascular invasion, and suitability for minimally invasive surgery. Patients with multifocal disease, posterior/superior lesions, or severe liver dysfunction were excluded. All patients underwent discussion in the hepatobiliary oncology team, and it was agreed upon that surgery would be an appropriate therapy according to the current practice guidelines [7]. All patients were operated on using the same surgical technique. The setup in the operating room is presented in Figure 1 [8].

In all procedures, a 4 cm peri-umbilical incision was made, through which the da Vinci SP Access Port© was introduced. Pneumoperitoneum was created at 12 mmHg, and the robotic setup was set up as per protocol. Additional trocar placements were performed when necessary.

Intra-operative ultrasound was always done at the start of the procedure in order to confirm the position of the tumor and set the borders for resection. Intracorporeal preparation for the Pringle maneuver was performed systematically to enable rapid and intermittent occlusion of the inflow in case it became necessary while dissecting the parenchyma. The liver was dissected using the MAMBA (Monopolar-Assisted Microwave-Based Ablation) technique, which allows for bloodless and controlled dissection by combining energy-based and mechanical methods of dissection [9].

The decision to place a surgical drain was left to the surgeon’s discretion and was based on intraoperative findings such as extent of parenchymal transection, risk of bile leakage, and intraoperative bleeding.

The clinical, intraoperative, and postoperative data were retrospectively obtained and retrospectively analyzed. Due to the small number of subjects, the continuous variables were presented as median and interquartile range (IQR), following the recommendations regarding the small cohorts [10].

All patients received a standardized multimodal analgesic protocol including paracetamol every 8 h. Pain was objectively measured by means of the Visual Analog Scale (VAS) and Numeric Rating Scale (NRS), which are recognized and well-established methods for measuring postoperative pain [11,12]. Pain scores were recorded postoperatively on day 0 and day 1.

## 3. Results

### 3.1. Patient Characteristics

In total, 11 consecutive patients were recruited. Baseline demographics are presented in Table 1.

The median age was 67 years (IQR 41–78), and the percentage of women was 63.6%. The median body mass index was 26.7 kg/m^2^ (IQR 22.5–32.2). The proportion of patients with ASA score ≥ III was 27.3%. Open and laparoscopic abdominal surgery had been previously performed by 27.3% and 18.2%, respectively. The incidence of cirrhosis was 27.3%; all cases had been assessed as Child–Pugh class A based on the original classification system [13]. No patients underwent neoadjuvant chemotherapy before the operation. The median serum albumin was 4.95 g/dL (IQR 3.9–5.5). Ca 19-9 and AFP were measured, and their median values were 7.5 U/mL (IQR 2.1–1160.6) and 5.1 ng/mL (IQR 2–503.5), respectively. The median tumor size was 25 mm (IQR 15–33); 27.3% of the tumors showed vascular contact. According to the Tampa classification for minimally invasive liver surgery (MILS), all operations were classified as difficulty grade II [14].

### 3.2. Intraoperative Outcomes

Intraoperative data are reported in Table 2.

**Figure 2:**
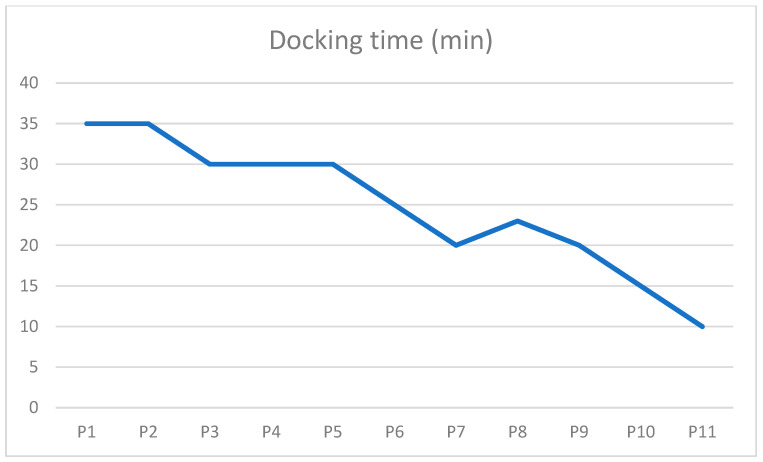
Trend in docking time over the learning curve.

Median operative time was 190 min (IQR 70–320), which included median docking time of 25 min (IQR 10–35) and console time of 165 min (IQR 50–290). A closer look at the docking time reveals that it has decreased over the course of the learning curve (Figure 2).

Assistant trocar use was needed for 45.4% of operations. Indications included liver retraction, suction, and assistance during parenchymal transection or stapling. In all cases, a single assistant trocar was placed.

Median intraoperative blood loss was 50 mL (IQR 0–300). Pedicle clamping was used in 36.4% of operations for a median time of 0 min (IQR 0–64). This result is due to the non-routine use of intermittent clamping, as it was not necessary in most patients; however, when applied, the duration reached up to 64 min. Surgical drainage was used in 63.6% of patients. As far as resection type is concerned, 2 operations (18.2%) were classified as anatomical resections, while 9 (81.8%) were non-anatomical resections according to the Brisbane 2000 terminology [15].

### 3.3. Postoperative Outcomes

Postoperative outcomes are summarized in Table 3.

The AST median value on day 1 was 81 U/L (IQR 20–542), ALT was 71 U/L (IQR 24–701), and total bilirubin was 0.8 mg/dL (IQR 0.4–4.3). It should be noted that the total bilirubin level of 4.3 mg/dL refers to a patient with Gilbert’s syndrome. The median values for prothrombin time and INR were 1.1 (IQR 0.9–1.24), whereas the median level of serum creatinine was 0.8 mg/dL (IQR 0.63–1.97). The pain on the postoperative day was low, with the median VAS score being 0 (IQR 0–2) on day 0 and 0 (IQR 0–3) on day 1. The median NRS score on day 0 was 0 (IQR 0–2) and on day 1 was 0 (IQR 0–3). The very low pain scores may be partially explained by early analgesic administration and the minimally invasive nature of the approach. The use of analgesics was stopped on postoperative day 1. Time to first flatus was 1 day in all cases. There were no postoperative complications. Postoperative ultrasonography was done in 45.4% of patients. The median length of hospital stay was 2 days and no patient required admission to the intensive care unit.

Final pathological examination confirmed preoperative diagnosis in all patients. Negative resection margins (R0), both parenchymal and vascular, were achieved in 100% of cases, with a median margin distance of 6 mm (IQR: 1–16).

## 4. Discussion

This article describes a pilot study of SP robotic liver resection in a homogenous group of patients undergoing MIS for liver lesions situated in anterolateral segments. This surgical technique may be viewed as an additional step in the field of MIS of the liver aimed at achieving a reduction in surgical invasiveness along with preserving all proven positive aspects of laparoscopic and robotic surgery.

MILS has been widely used in the last two decades due to the evidence confirming better outcomes of this procedure compared to open surgery. Specifically, MILS is associated with less blood loss, lower incidence of complications, shorter hospitalization, and faster return of normal functional status [16,17]. Indications for MILS have been gradually expanding during international conferences [14,16].

Another factor that has driven this process is the application of robotic platforms, which have helped to overcome some limitations inherent to laparoscopic surgery, including limited instrument mobility, unsteady camera support, and ergonomic issues [18,19]. Robotic technology has the advantage of greater dexterity, tremor damping, and three-dimensional vision, and it may allow performing even complex liver resection [18,19]. Recently, the emergence of specialized SP robotic platforms has offered new possibilities in MIS, as this approach aims to reduce injury to the abdominal wall while maintaining high precision during the procedure.

However, despite all these theoretical benefits, there is not enough evidence regarding SP robotic surgery of the liver. The majority of the existing experience is based on small case series that differ from each other by various factors, such as the degree of heterogeneity in patient selection, tumor characteristics, and complexity of the procedure performed. Therefore, the current paper adds another piece of information concerning SP robotic liver surgery by describing cases with relatively low complexity, as evidenced by the consistent difficulty grade II of all procedures according to the Tampa scale [14].

Technically speaking, the results of this study suggest the feasibility of the SP robotic approach in carefully selected patients. Despite the fact that an additional trocar for an assistant was necessary in some cases, it was expected due to the novelty of the approach, and it can be considered a part of its implementation period or the lack of staplers or advanced energy sources compatible with SP. The operation time in this case seems to correspond to the published data on robotic liver resection, taking into account the impact of the learning curve for both robotic and SP techniques [18,20].

Bleeding during the procedure was minimal, and pedicle clamping was applied only where needed according to the existing practice in MILS to balance hemostasis and preserve the function of the organ [16]. This result corresponds to the existing publications on the safety of robotic liver resection regarding intraoperative hemodynamic control.

The postoperative results were quite promising for this group of patients. There were no complications or reinterventions documented, and the duration of hospitalization was very short. Additionally, the level of postoperative pain was minimal, as well as the usage of pain medications and fast recovery of bowel function. All of these findings are consistent with the expected benefits from minimally invasive techniques and could indicate a possible application of the SP approach in improving postoperative outcomes; however, the mentioned conclusions should be drawn cautiously.

There are certain drawbacks to the current research that need to be mentioned. First of all, the sample size of the study is a limitation because of its small size. Only 11 patients were included into the study; thus, it is statistically insignificant and can vary due to chance. It means that even the lack of observed complications cannot be used as proof of safety, since rare or infrequent complications might have occurred but not be observed in such a small sample.

Second, there are several biases that could potentially arise from the retrospective nature of the study. The selection of patients was not random; furthermore, the inclusion of highly selected cases, namely lesions in anterolateral segments categorized as Tampa grade II, reduces the generalizability of the findings. The use of such patients for this kind of study is justified; however, it limits the ability to generalize the findings to more difficult operations or different types of patients, for example, patients with severe liver disease or more challenging tumors.

Third, the lack of a control group makes any comparative analysis impossible. It is not possible to compare the results achieved by using the SP method with those obtained with multiport robotic or laparoscopic procedures. Therefore, the study cannot be regarded as a comparative study, and no conclusion can be drawn about the effectiveness of this approach in comparison with others.

Another limitation pertinent to the paper under discussion concerns the learning curve. The implementation of SP robotic surgery implies quite a challenging shift even for surgeons who perform MILS. The time needed to perform the surgery, number of trocars required, and other aspects can be affected by the initial stage of the learning curve. However, the current study does not consider the issue, which means that it is difficult to differentiate between results caused by the technique itself and those dependent on the surgeon’s experience.

Finally, there is no information about the oncological outcomes. While the size, main features of tumors, margins of resection and R0 parenchymal and vascular rates are described, there are no details concerning frequency of recurrences, and survival rate. This limitation is especially important in terms of liver surgery when oncological adequacy is of crucial importance. There are no follow-up data that could shed light on the issue.

Another aspect that should be mentioned is the heterogeneity related to some perioperative management measures like the application of pedicle clamping or postoperative imaging. These parameters are reported in the paper, but their heterogeneity might be due to different decisions made during the surgery rather than any protocol, thus complicating the research even more.

Last but not least, the study lacks patient-reported outcomes and cost analysis. These parameters are considered vital when assessing new surgical approaches, especially minimally invasive procedures. This means that the current study provides an incomplete picture.

Given the above-mentioned limitations, the results of the paper can hardly be considered conclusive.

## 5. Conclusions

In the current pilot series, it was proven that single-port robotic liver resection is technically possible in a very selective population of patients with tumors situated in anterolateral segments and characterized as intermediate difficulty by the Tampa classification system [14]. This method is linked to good results from both an intraoperative and early postoperative point of view, which include minimal blood loss, no complications, and fast recovery.

Nonetheless, due to the small number of patients involved, retrospective character, and lack of a control group, these results must be taken cautiously into account. More research is needed to clarify the place of single-port robotic liver surgery in the current spectrum of minimally invasive approaches to liver surgery.

## Figures and Tables

**Table 1:** Preoperative data.

Variables	N = 11
Age (year), median	67 (IQR: 41–78)
Female, n (%)	7 (63.6%)
BMI (kg/m^2^), median	26.7 (IQR: 22.5–32.2)
ASA score ≥ III, n (%)	3 (27.3%)
Previous open abdominal surgery, n (%)	3 (27.3%)
Previous laparoscopic abdominal surgery, n (%)	2 (18.2%)
Cirrhosis, n (%)	3 (27.3%)
Serum albumin (g/dL), median	4.95 (IQR: 3.9–5.5)
Ca 19.9 (U/mL), median	7.5 (IQR: 2.1–1160.6)
AFP (ng/mL), median	5.1 (IQR: 2–503.5)
Tumor size, median	25 (IQR: 15–33)
Vascular contact, n (%)	3 (27.3%)

BMI: Body Mass Index; ASA: American Society of Anesthesiologists; AFP: Alpha-fetoprotein.

**Table 2:** Intraoperative data.

Variables	N = 11
Total operative time (min), median	190 (IQR: 70–320)
Docking time (min), median	25 (IQR: 10–35)
Console time (min), median	165 (IQR: 50–290)
Extra assistant trocar, n (%)	5 (45.4%)
Intraoperative blood loss (mL), median	50 (IQR: 0–300)
Pedicle clamping, n (%)	4 (36.4%)
Pedicle clamping duration (min), median	0 (IQR: 0–64)
Drain, n (%)	7 (63.6%)

**Table 3:** Postoperative data.

Variables	N = 11
POD 1 AST (U/L), median	81 (IQR: 20–542)
POD 1 ALT (U/L), median	71 (IQR: 24–701)
POD 1 BILT (mg/dL), median	0.8 (IQR: 0.4–4.3)
POD 1 TP, median	1.1 (IQR: 0.9–1.24)
POD 1 INR, median	1.1 (IQR: 0.9–1.24)
POD 1 Serum creatinine (mg/dL), median	0.8 (IQR: 0.63–1.97)
VAS POD 0, median	0 (IQR: 0–2)
VAS POD 1, median	0 (IQR: 0–3)
NRS POD 0, median	0 (IQR: 0–2)
NRS POD 1, median	0 (IQR: 0–3)
Post-operative complication, n (%)	0 (0%)
Post-operative US, n (%)	5 (45.4%)
Total hospital stay (day), median	2 (IQR: 1–3)
Reintervention, n (%)	0 (0%)
90-day readmission, n(%)	0 (0%)
R0 parenchymal, n (%)	0 (100%)
R0 vascular, n (%)	0 (100%)
Margin distance (mm), median	6 (IQR: 1–16)

POD: Post-Operative Day; VAS: Visual Analogue Scale; NRS: Numerical Rating Scale; US: Ultrasonography.

## Data Availability

The data presented in this study are available upon request from the corresponding author due to privacy reasons.
